# Whole-body heat treatment stimulates antigen-specific T cell responses in human system

**DOI:** 10.1186/2051-1426-1-S1-P131

**Published:** 2013-11-07

**Authors:** Yasunobu Kobayashi, Yusuke Ito, Mayuko Sakai, Masanori Matsushita, Kenichiro Imai, Koichi Shimizu, Atsushi Aruga, Keishi Tanigawa

**Affiliations:** 1 Bio-Thera Clinic, Tokyo, Japan; 2 Institute of Gastroenterology, Tokyo Women's Medical University, Tokyo, Japan; 3 Shin-Itabashi Clinic, Tokyo, Japan; 4 Institute of Advanced BioMedical Engineering and Science, Tokyo Women's Medical University, Tokyo, Japan

## 

Previous studies in mice have showed that fever-range thermal stress (approx. 38.5-40.5 degrees C) stimulates a variety of T cell functions ex vivo and in vivo. The aim of this study is to examine the effects of increase in core body temperature in human system on the function of peripheral T cells, and in particular their IFNγ production in response to specific antigens. Using the Heckel ht-3000, a device specifically designed for physiological range whole-body hyperthermia, healthy volunteers were heated until the rectal temperature reached at 38.5 degrees C. They were then wrapped in synthetic leather tent for 60 min in order to maintain the core body temperature above 38.5 degrees C. Peripheral blood was obtained four times; prior to the treatment, immediately after, 24 h after and 48 h after the treatment. PBMCs were then prepared and co-cultured with antigen-loading autologous monocyte-derived dendritic cells (DCs) for 24 h in order to induce antigen specific IFNγ productions in T cells. We first examined the IFNγ production in T cells in response to MHC class I restricted epitope peptides of CMV, EBV and Flu. When PBMCs were co-cultured with these peptides-labeled DCs, a marked increase in IFNγ production was observed in PBMCs prepared immediately after and 24 h after the whole-body heat treatment. Production of IFNγ in PBMCs prepared 48 h after the treatment returned to approximately the same level as in those without the treatment. Similarly, enhanced production of IFNγ in response to the tuberculin purified protein derivative was also observed immediately after and 24 h after the treatment. In fluorescence photobleaching analyses, we found that the rate of fluorescence recovery after photobleaching was accelerated in T cells prepared both immediately after and 24 h after the heat treatment, suggesting that the increase in cell membrane fluidity induced by heat treatment could be one of the possible mechanisms to stimulate T cell functions in response to specific antigens. Taken together, we conclude that whole-body heat treatment in physiological range stimulates antigen-specific T cell responses, and thus it could be a possible combination therapy to enhance the efficacy of cancer immuno cell therapy, such as adoptive transfer of activated T cells and DCs-based vaccination.

